# Supplementary material for: Amplification of USP13 drives ovarian cancer metabolism
Source: Nat Commun. 2016 Nov 28;7:13525. doi: 10.1038/ncomms13525 (PMC5133706; doi:10.1038/ncomms13525)
Supplement: Supplementary Information — Supplementary Figures 1-11. [file ncomms13525-s1.pdf]

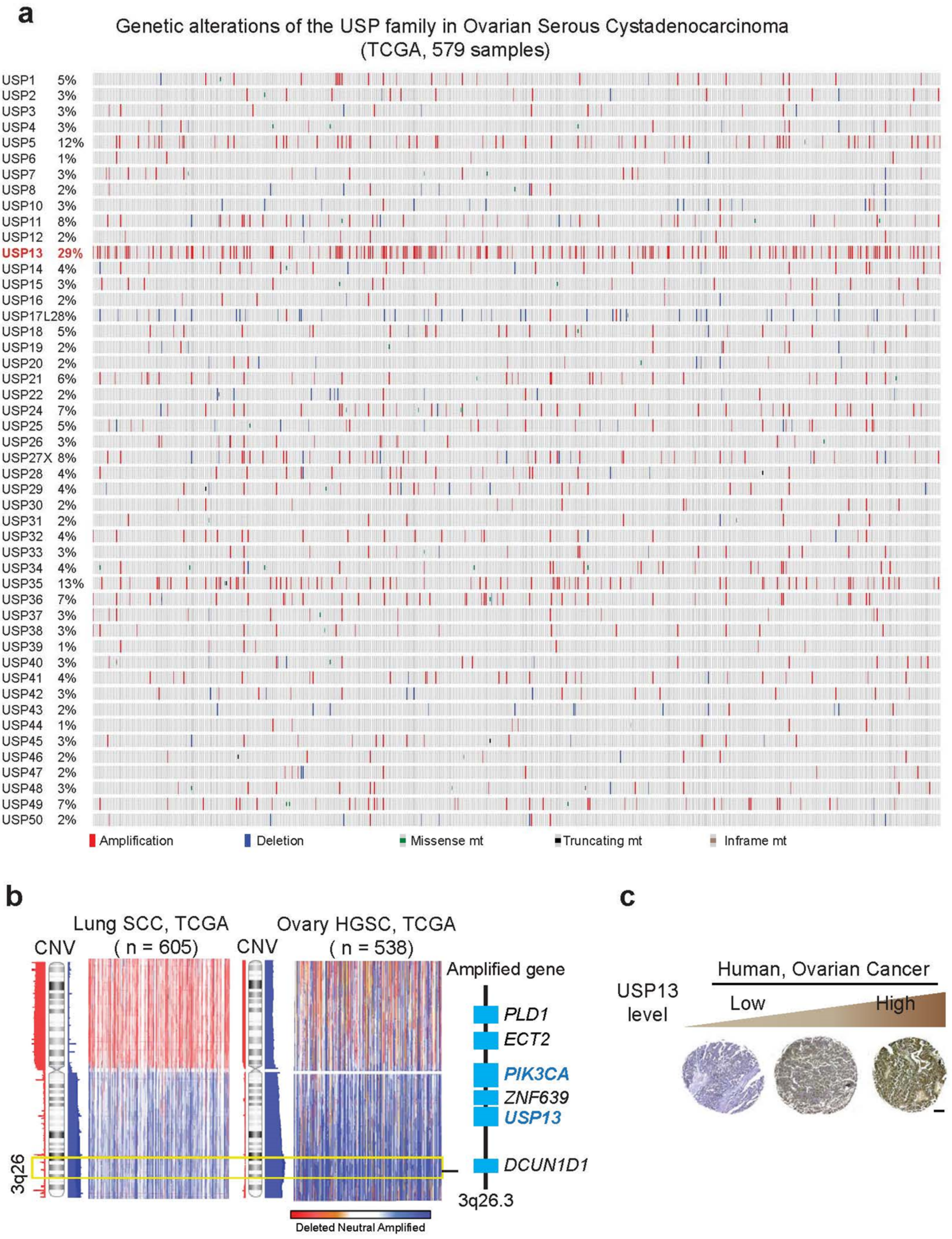
a
Genetic alterations of the USP family in Ovarian Serous Cystadenocarcinoma
(TCGA, 579 samples)
USP1 5%
USP2 3%
USP3 3%
USP4 3%
USP5 12%
USP6 1%
USP7 3%
USP8 2%
USP10 3%
USP11 8%
USP12 2%
USP13 29%
USP14 4%
USP15 3%
USP16 2%
USP17L28%
USP18 5%
USP19 2%
USP20 2%
USP21 6%
USP22 2%
USP24 7%
USP25 5%
USP26 3%
USP27X 8%
USP28 4%
USP29 4%
USP30 2%
USP31 2%
USP32 4%
USP33 3%
USP34 4%
USP35 13%
USP36 7%
USP37 3%
USP38 3%
USP39 1%
USP40 3%
USP41 4%
USP42 3%
USP43 2%
USP44 1%
USP45 3%
USP46 2%
USP47 2%
USP48 3%
USP49 7%
USP50 2%
Amplification
Deletion
Missense mt
Truncating mt
Inframe mt
b
Lung SCC, TCGA
(n = 605)
CNV
Ovary HGSC, TCGA
(n = 538)
CNV
Amplified gene
PLD1
ECT2
PIK3CA
ZNF639
USP13
DCUN1D1
3q26
3q26.3
Deleted Neutral Amplified
c
Human, Ovarian Cancer
USP13 level
Low
High

Supplementary Figure 1. Genomic amplification and aberrant expression of USP13 in OVCAs. (**a**) Genomic alterations of USP family members in ovarian serous adenocarcinomas determined by cBioPortal analysis of TCGA databases. (**b**) Integrated analysis of USP13 amplification in human lung and ovarian cancers. Frequency plots of the copy-number abnormalities indicate degree of copy number loss (red) or gain (blue). (**c**) Scoring of USP13 signals in ovarian adenocarcinoma. Scoring = intensity (1, 2 or 3) x proportion (10-100 %) (Scale bar: 100 μm).

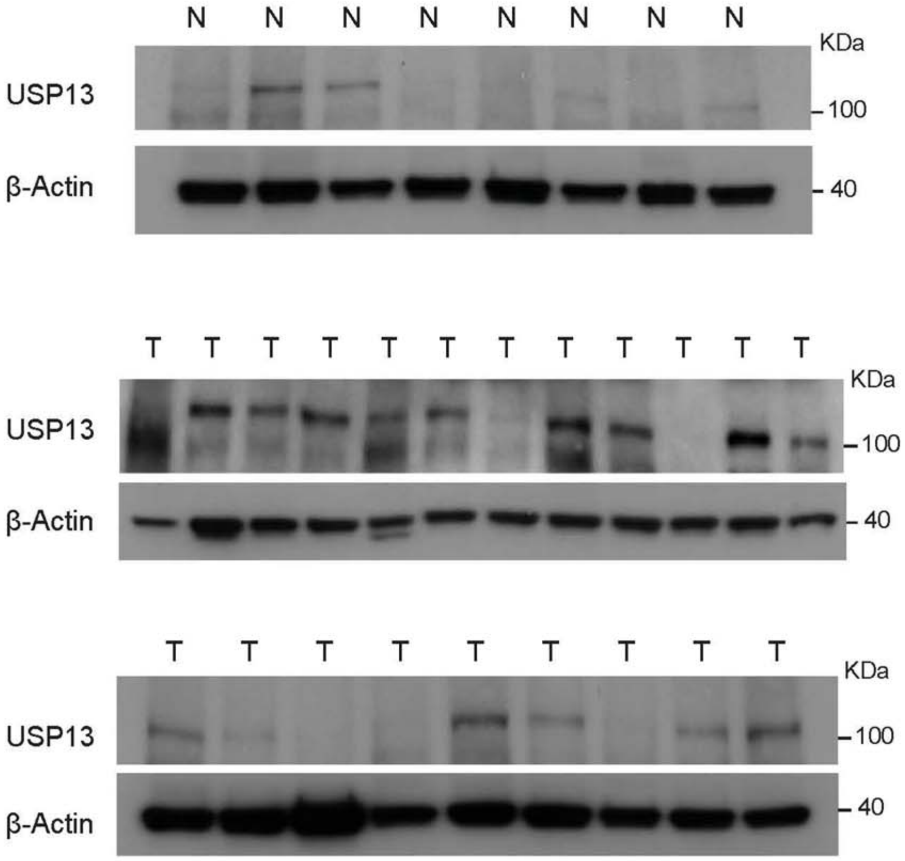

Supplementary Figure 2. Western blot analysis of USP13 levels in human normal ovarian tissue (N, 8) and ovarian tumour tissue (T, 21) samples. β-Actin was used as a loading control.

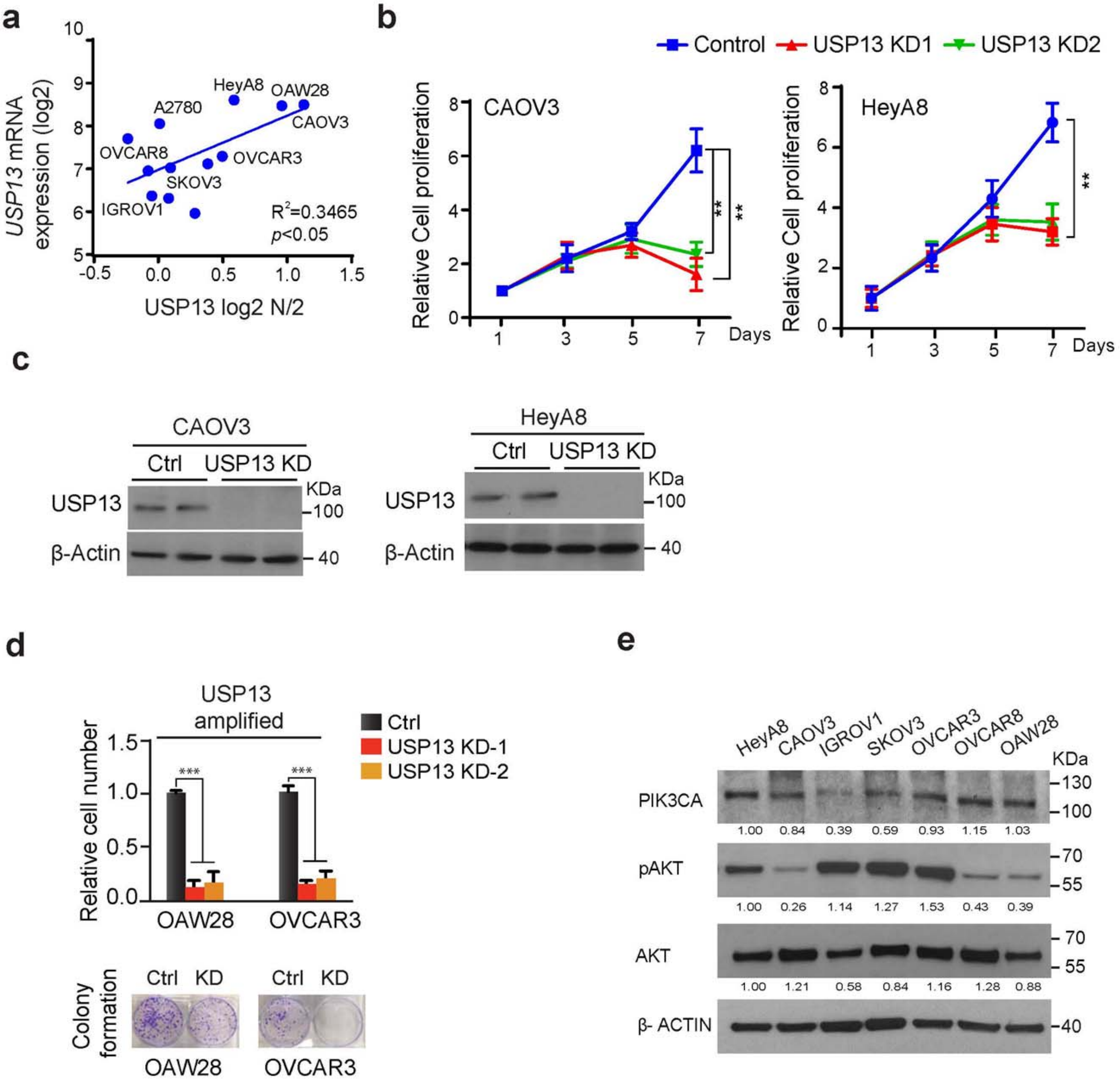

Supplementary Figure 3. Inhibiting USP13 suppresses OVCA cell proliferation. (**a**) A plot of USP13 mRNA expression versus copy number in representative ovarian cancer cell lines. Linear regression with an R^2=0.3465. (**b**) Relative cell proliferation of CAOV3 and HeyA8 cells transduced with lentiviral control or USP13 shRNAs (KD1 or 2; KD: knockdown). Error bars represent ± SD. **p<0.01. (**c**) Western blot analysis of USP13 levels in control or USP13-knockdown (KD) CAOV3 and HeyA8 cells. β-Actin was used as a loading control. (**d**) Relative proliferation of OAW28 and OVCAR3 cells stably expressing control or USP13 shRNAs. The non-silencing scramble shRNA was used as a negative control. After 7 days treatment of shControl or shUSP13, crystal violet staining of cells were performed. (**e**) Expression of PIK3CA, phospho-AKT1 (S473), total AKT and β-Actin were analysed in ovarian cell lines. Protein blots were quantified and normalized to that of β-Actin.

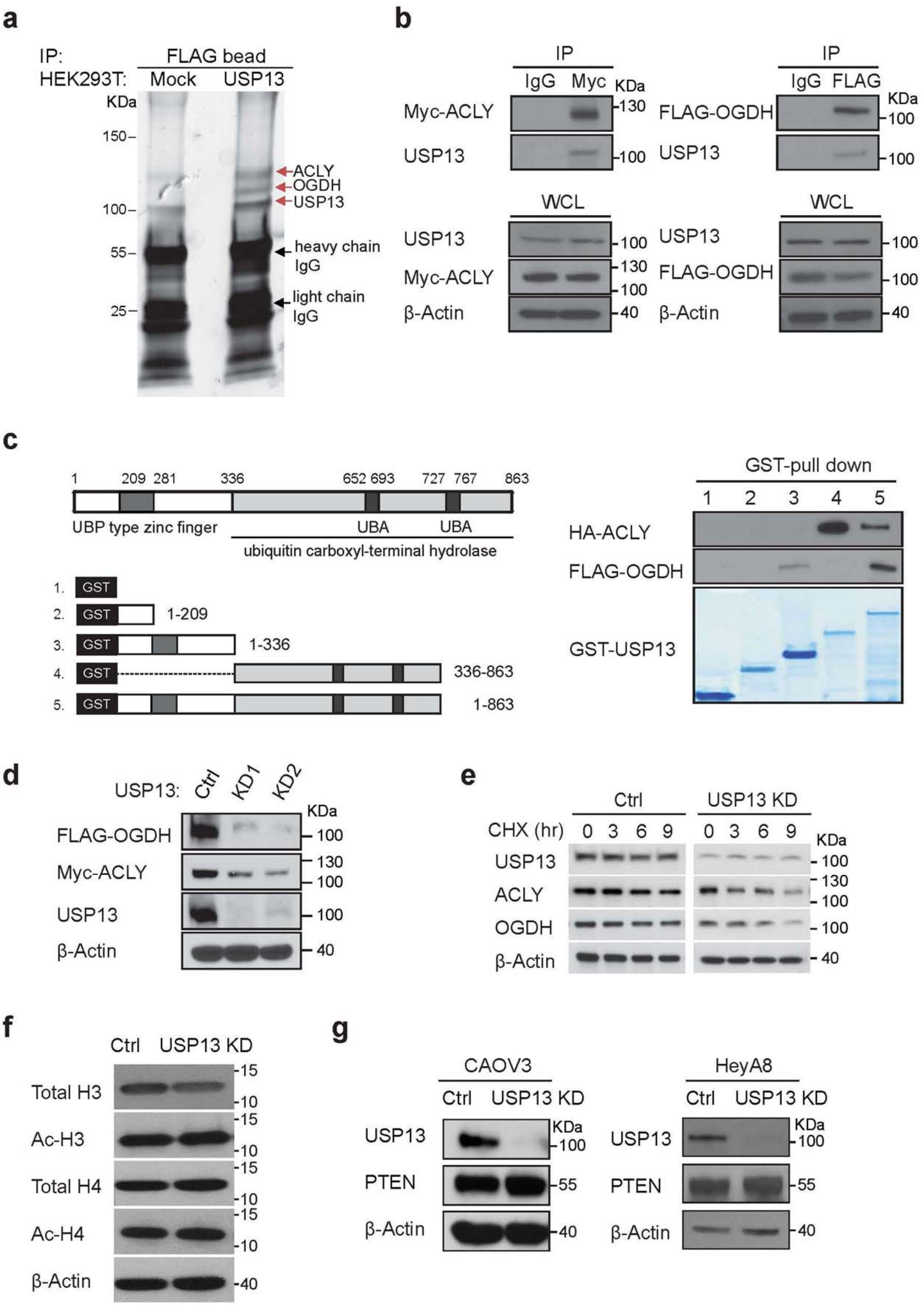

a
IP:
FLAG bead
HEK293T:
Mock
USP13
KDa
150
100
55
25
ACLY
OGDH
USP13
heavy chain
IgG
light chain
IgG
b
IP
IgG
Myc
KDa
Myc-ACLY
USP13
130
100
WCL
USP13
Myc-ACLY
β-Actin
100
130
100
40
IP
IgG
FLAG
KDa
FLAG-OGDH
USP13
100
100
WCL
USP13
FLAG-OGDH
β-Actin
100
100
40
c
1
209
281
336
652
693
727
767
863
UBP type zinc finger
UBA
UBA
ubiquitin carboxyl-terminal hydrolase
1. GST
2. GST 1-209
3. GST 1-336
4. GST 336-863
5. GST 1-863
GST-pull down
1
2
3
4
5
HA-ACLY
FLAG-OGDH
GST-USP13
d
USP13:
Ctrl
KD1
KD2
KDa
FLAG-OGDH
Myc-ACLY
USP13
β-Actin
100
130
100
100
40
e
Ctrl
USP13 KD
CHX (hr)
0
3
6
9
0
3
6
9
KDa
USP13
ACLY
OGDH
β-Actin
100
130
100
100
40
f
Ctrl
USP13 KD
Total H3
Ac-H3
Total H4
Ac-H4
β-Actin
15
10
15
10
15
10
15
10
40
g
CAOV3
Ctrl
USP13 KD
USP13
PTEN
β-Actin
KDa
100
55
40
HeyA8
Ctrl
USP13 KD
USP13
PTEN
β-Actin
KDa
100
55
40

Supplementary Figure 4. USP13 physically interacts with ACLY and OGDH. (**a**) Silver staining of USP13-containing immunoprecipitates purified from control or USP13-overexpressing HEK293T cells. (**b**) Exogenously expressed Myc-ACLY or FLAG-OGDH interacts with endogenous USP13 in HEK293T cells. (**c**) Schematic representation of USP13 domains and truncated forms of USP13 (left panel). GST-fused USP13 proteins were immobilized on glutathione-Sepharose beads and mixed with the lysates of HEK293T cells expressing HA-ACLY or FLAG-OGDH. Immunoprecipitates were examined with anti-HA or anti-FLAG antibodies (right panel). (**d**) Immunoblotting analysis shows that USP13 knockdown decreases the levels of exogenously expressed FLAG-OGDH and Myc-ACLY. (**e**) USP13 knockdown decreases the protein stability of ACLY and OGDH in CAOV3 cells treated with cycloheximide. CAOV3 cells expressing control or USP13 shRNA were treated with cycloheximide (CHX, 100 μg ml^{-1}) for indicated time points. (**f**) Immunoblotting analysis of total Histone 3 (H3), acetyl histones H3 (H3Ac), total histone 4 (H4) and acetyl histone H4 (H4Ac) were performed in control and USP13-knockdown CAOV3 cells. (**g**) PTEN levels were examined in control and USP13-knockdown CAOV3 and HeyA8 cell lines.

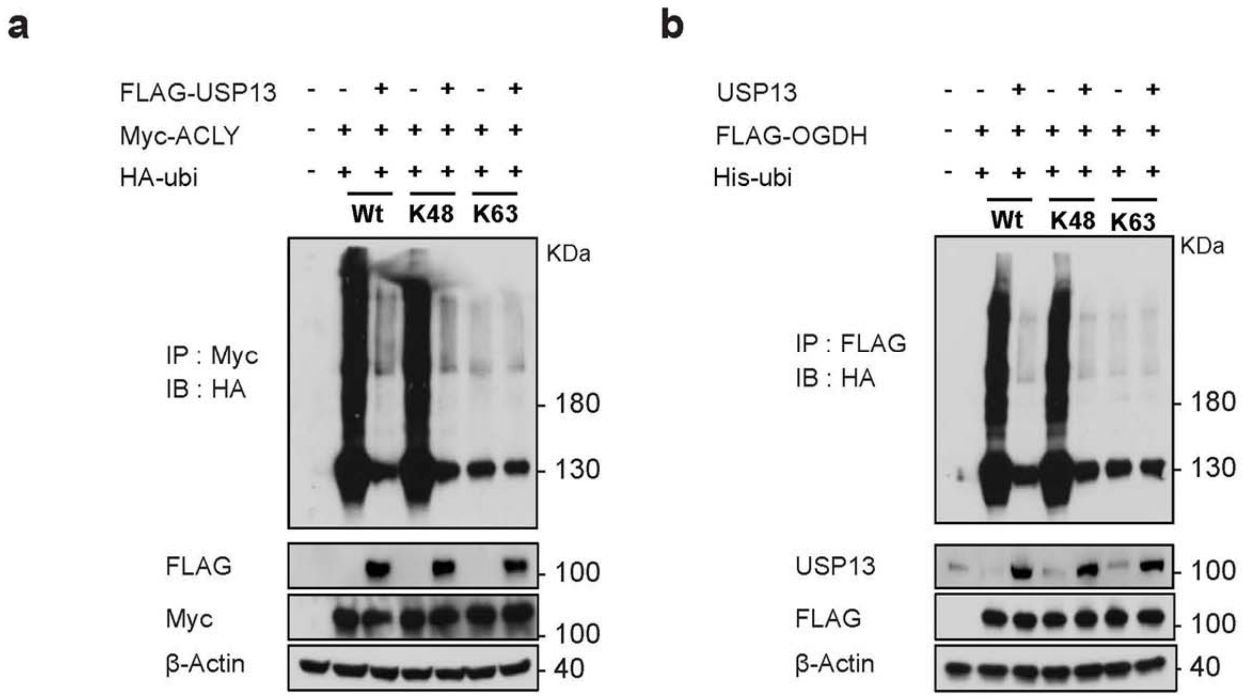

Supplementary Figure 5. USP13 removes K48-linked ubiquitination of ACLY (**a**) and OGDH (**b**). HEK293 cells were co-transfected with indicated expression vectors. K48: K48-only ubiquitin, and K63: K63-only ubiquitin. Equal amounts of cell lysates were analysed by immunoprecipitation (IP) and immunoblotting (IB) assays as indicated.

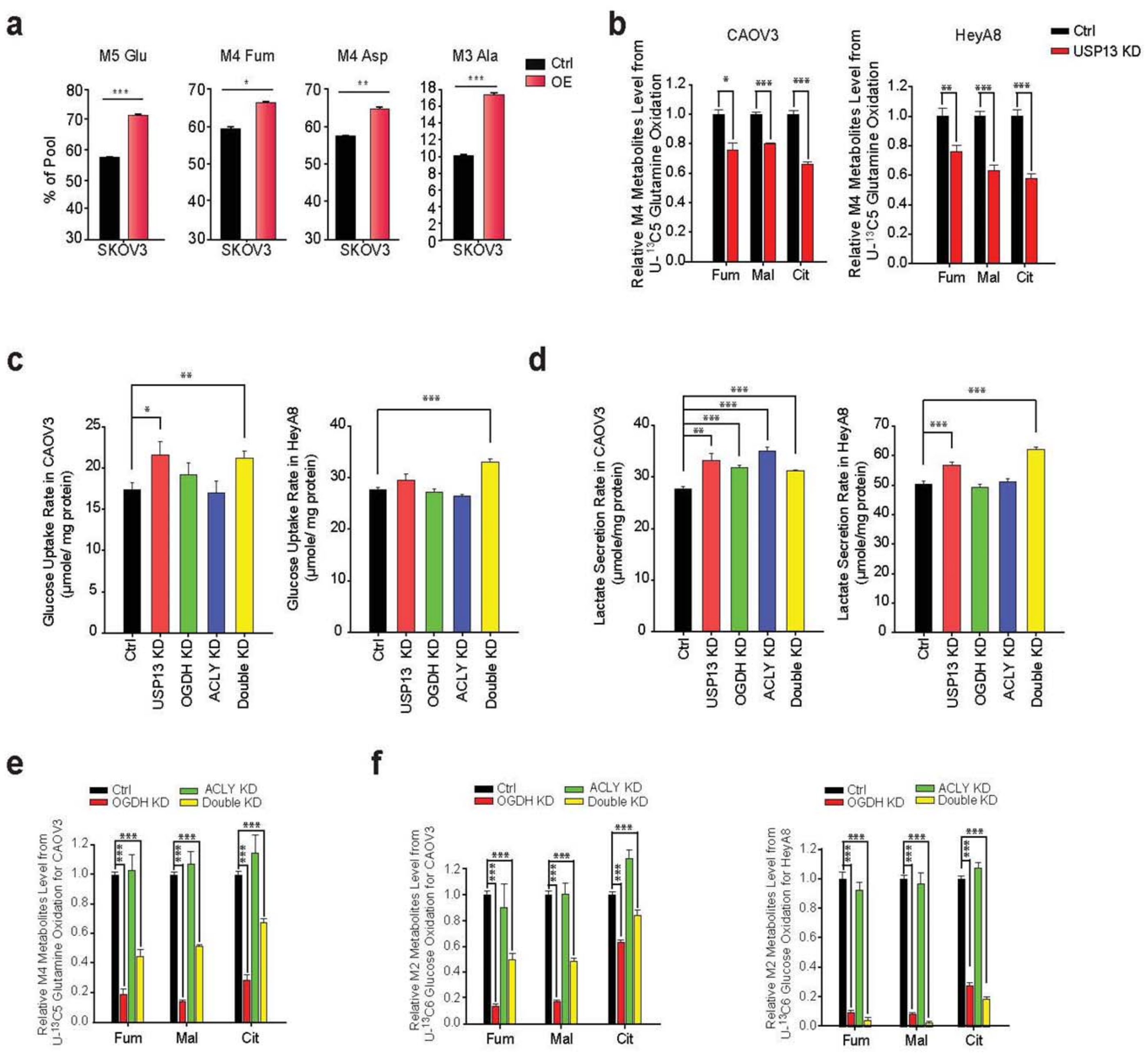

Supplementary Figure 6. USP13 maintains the metabolite pools in TCA cycle by regulating OGDH expression. (**a**) Contribution of glutamine to TCA metabolites and glutamate pool in control and USP13-overexpressing (OE) SKOV3 cells. (**b**) Relative levels of M4 fumarate, malate and citrate that are derived from [U-^{13}C] glutamine (Gln) in control and USP13-knockdown (KD) CAOV3 and HeyA8 cells. (**c**, **d**) Effect of USP13 knockdown, OGDH knockdown, ACLY knockdown, and OGDH/ACLY double knockdown on the glucose uptake rate (c) and lactate secretion rate (d) in CAOV3 and HeyA8 cells. (**e**) Relative levels of M4 fumarate, malate and citrate that are derived from [U-^{13}C] Gln in control, OGDH-knockdown, ACLY-knockdown, and OGDH/ACLY-double knockdown CAOV3 cells. (**f**) Relative levels of M2 fumarate, malate, and citrate that are derived from [U-^{13}C] glucose (Glc) in control, OGDH-knockdown, ACLY-knockdown, and OGDH/ACLY-double knockdown CAOV3 and HeyA8 cells. In this figure, error bars represent ± SEM. *p<0.05, ** p<0.01, *** p<0.001.

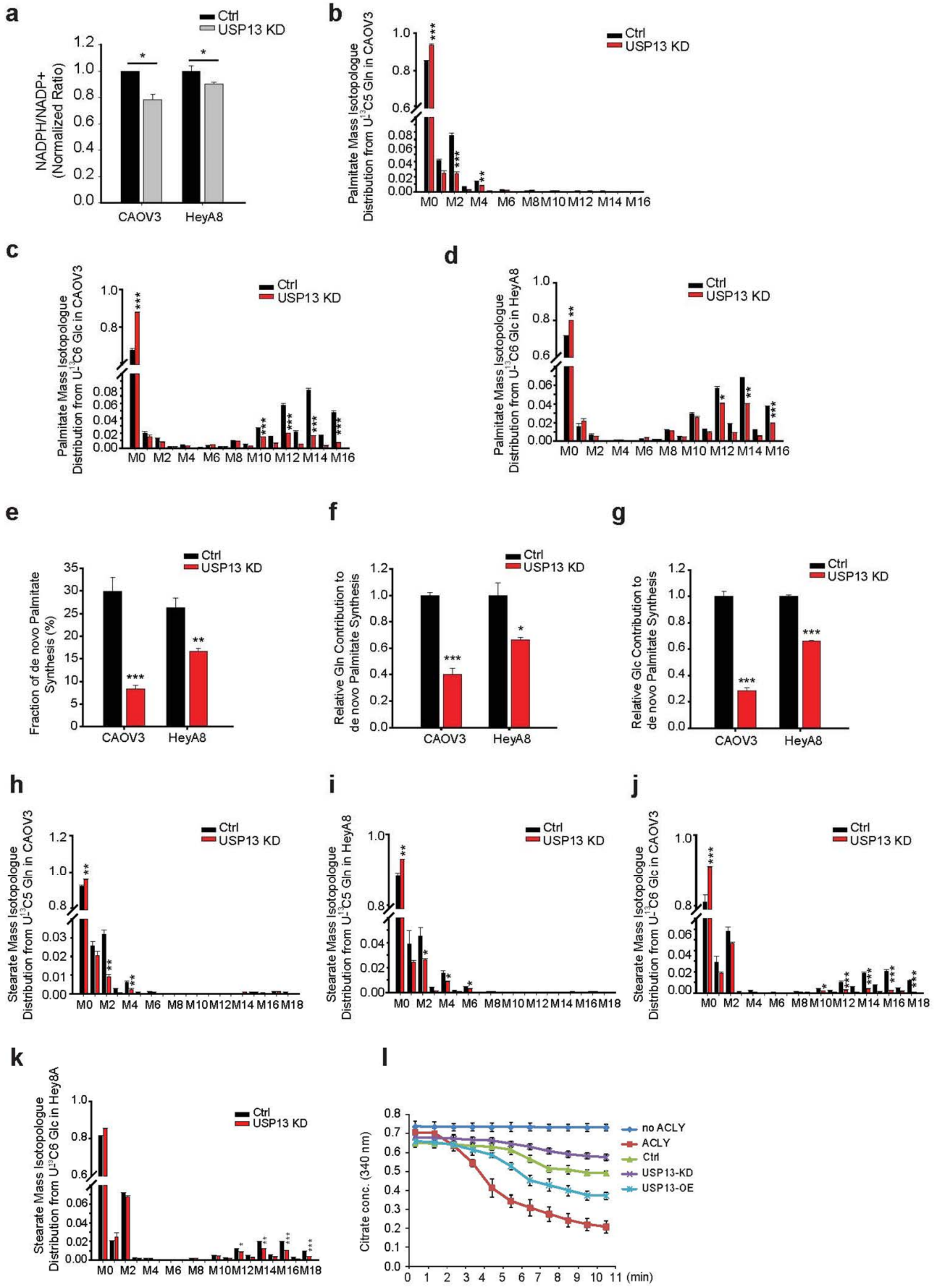

a
Ctrl
USP13 KD
NADPH/NADP+ (Normalized Ratio)
CAOV3
HeyA8
b
Palmitate Mass Isotopologue Distribution from U-13C5 Gln in CAOV3
Ctrl
USP13 KD
M0 M2 M4 M6 M8 M10 M12 M14 M16
c
Palmitate Mass Isotopologue Distribution from U-13C6 Glc in CAOV3
Ctrl
USP13 KD
d
Palmitate Mass Isotopologue Distribution from U-13C6 Glc in HeyA8
Ctrl
USP13 KD
e
Fraction of de novo Palmitate Synthesis (%)
Ctrl
USP13 KD
CAOV3
HeyA8
f
Relative Gln Contribution to de novo Palmitate Synthesis
Ctrl
USP13 KD
g
Relative Glc Contribution to de novo Palmitate Synthesis
Ctrl
USP13 KD
h
Stearate Mass Isotopologue Distribution from U-13C5 Gln in CAOV3
Ctrl
USP13 KD
M0 M2 M4 M6 M8 M10 M12 M14 M16 M18
i
Stearate Mass Isotopologue Distribution from U-13C5 Gln in HeyA8
Ctrl
USP13 KD
j
Stearate Mass Isotopologue Distribution from U-13C6 Glc in CAOV3
Ctrl
USP13 KD
k
Stearate Mass Isotopologue Distribution from U-13C6 Glc in Hey8A
Ctrl
USP13 KD
l
Citrate conc. (340 nm)
(min)
no ACLY
ACLY
Ctrl
USP13-KD
USP13-OE

Supplementary Figure 7. Loss of USP13 reduces glutamine-driven de novo fatty acids synthesis. (**a**) Effect of USP13 knockdown on NADPH/NADP+ ratio in CAOV3 and Hey8A cells. (**b**) Labeling of palmitate in control and USP13-knockdown HeyA8 cells cultured for 72 h in the medium containing U-^{13}C glutamine (Gln). (**c**) Labeling of palmitate in control and USP13-knockdown CAOV3 cells cultured for 72 h in the medium containing U-^{13}C Gln. (**d, e**) Labeling of palmitate in control and USP13-knockdown CAOV3 (d) and HeyA8 (e) cells cultured for 72 h in the medium containing U-^{13}C glucose (Glc). (**f**) Isotopologue spectral analysis (ISA) of de novo palmitate synthesis in USP13-knockdown cells. (**g, h**) Relative glucose (g) or glutamine (h) contribution towards de novo synthesized palmitate in control and USP13-knockdown CAOV3 and Hey8A. (**i, j**) Labeling of stearate in control and USP13 knockdown CAOV3 (i) and HeyA8 (j) cells cultured for 72 h in the medium containing U-^{13}C Gln. (**k, l**) Labeling of stearate in control and USP13 knockdown CAOV3 (k) and HeyA8 (l) cells cultured for 72 h in the medium containing U-^{13}C Glc. (**m**) Enzymatic activity of ACLY was measured using purified ACLY protein and ACLY-containing cell lysates from USP13 knockdown and USP13-OE cells. In this figure, error bars represent ± SEM. *p<0.05, **p<0.01, ***p<0.001.

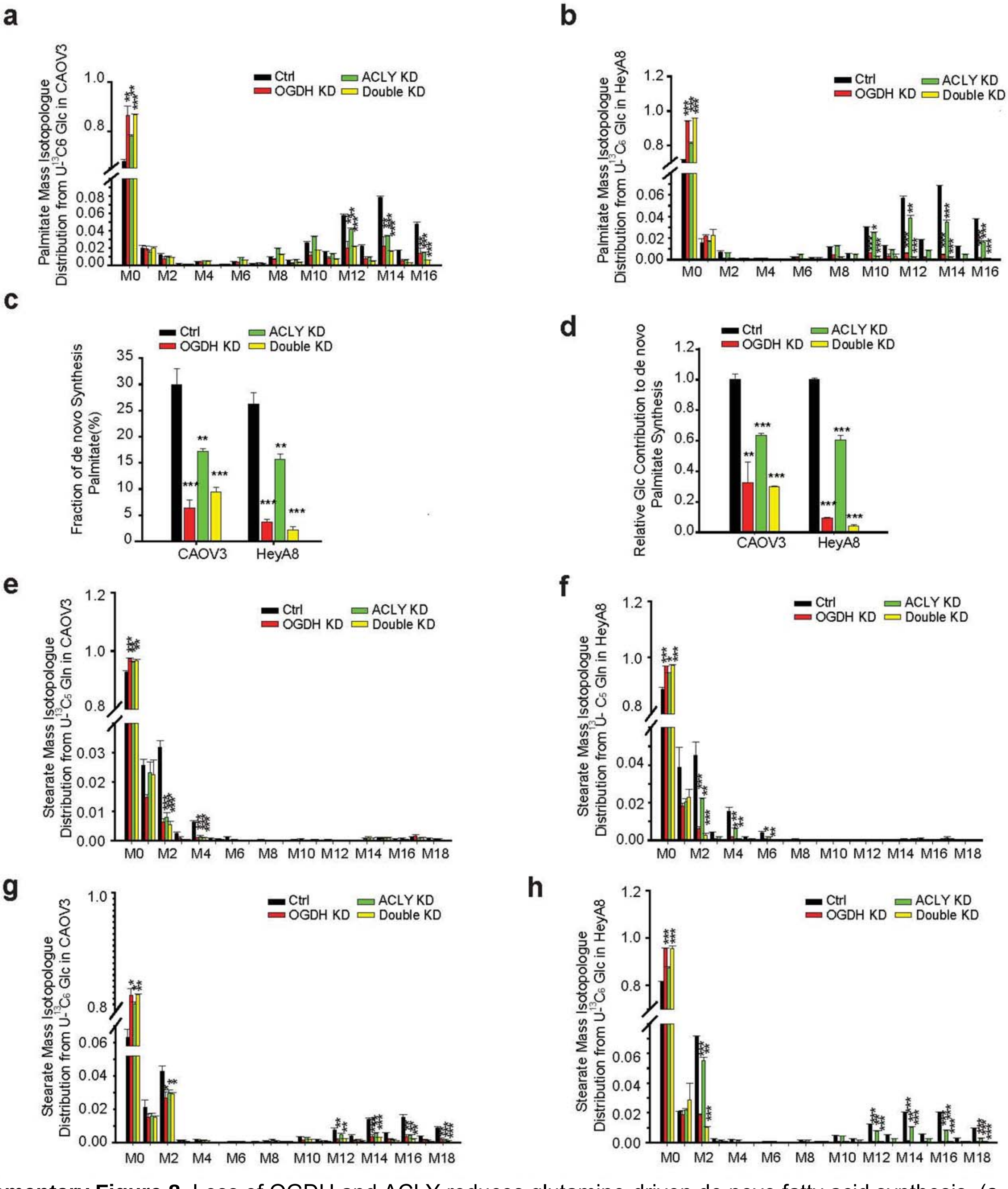

Supplementary Figure 8. Loss of OGDH and ACLY reduces glutamine-driven de novo fatty acid synthesis. (**a, b**) Labeling of palmitate in control, OGDH-knockdown, ACLY-knockdown, OGDH/ACLY-double knockdown CAOV3 (a) and HeyA8 (b) cells cultured for 72 h in the medium containing U-^{13}C glucose (Glc). (**c**) Isotopologue spectral analysis (ISA) of lipid synthesis in control, OGDH-knockdown, ACLY-knockdown and OGDH/ACLY-double knockdown CAOV3 and HeyA8 cells. (**d**) Relative glucose contribution towards de novo palmitate synthesis in control, OGDH-knockdown, ACLY-knockdown and OGDH/ACLY-double knockdown CAOV3 and Hey8A cells. (**e, f**) Labeling of stearate in control, OGDH-knockdown, ACLY-knockdown and OGDH/ACLY-double knockdown CAOV3 (e) and HeyA8 (f) cells cultured for 72 h in the medium containing U-^{13}C Gln. (**g, h**) Labeling of stearate in control, OGDH-knockdown, ACLY-knockdown and OGDH/ACLY-double knockdown CAOV3 (g) and HeyA8 (h) cells cultured for 72 h in the medium containing U-^{13}C Glc. In this figure, error bars represent ± SEM. **p*<0.05, ***p*<0.01, ****p*<0.001.

a

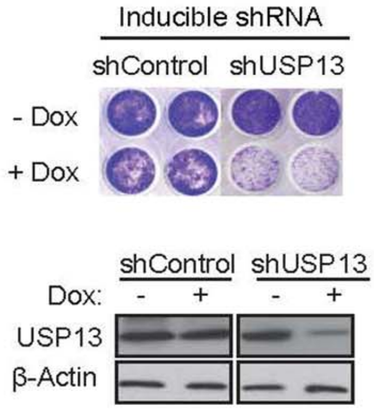

b

	Mice	Mesentary	Omentum	Diaphragm	Peritoneal wall	Perihepatic	Pelvic cavity	Others
Dox -	N+/N1	10/10	9/10	6/10	9/10	5/10	8/10	3/10
Dox +	N+/N1	3/10	4/10	0/10	3/10	0/10	2/10	0/10
Fisher's exact test	*P* value	0.0031	0.0573	0.0108	0.0198	0.0325	0.0230	0.2105

c

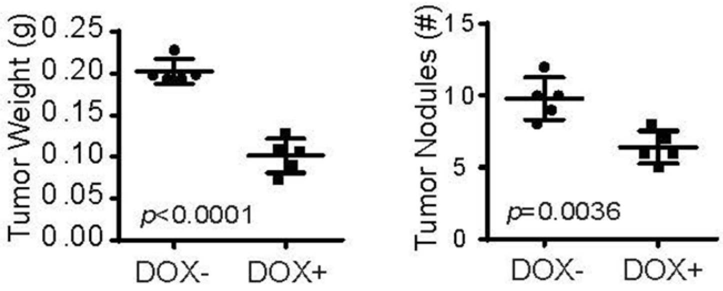

Supplementary Figure 9. Inhibiting USP13 suppresses ovarian tumour growth. (**a**) Dox-inducible knockdown of USP13 inhibited the proliferation of CAOV3 cells in vitro. (**b**) Metastatic sites and frequency of ovarian tumour derived from control and USP13-knockdown CAOV3 cells. Other sites include paraaortic lymph nodes, kidney, and liver. N+, number of mice harbouring positive tumour lesion; N1, total number of mice in Doxycycline non-treated (-) or treated (+) group. Significant difference in the metastatic patterns of these two groups were compared by Fisher's exact test. (**c**) Tumour weights and nodule numbers of the SKOV3-derived ovarian tumours expressing Dox-inducible USP13 shRNA.

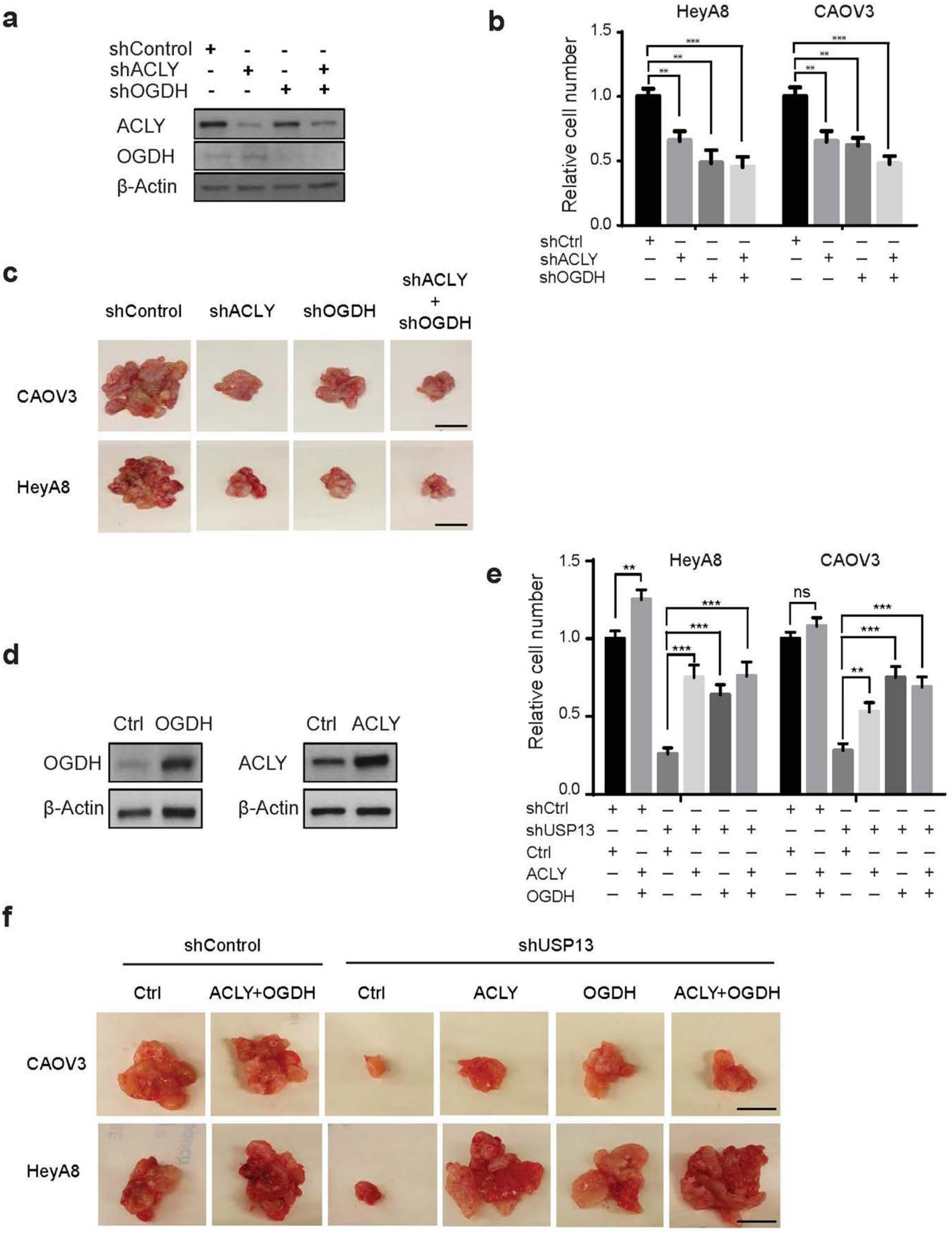
a
shControl + - - -
shACLY - + - +
shOGDH - - + +
ACLY
OGDH
β-Actin
b
HeyA8
CAOV3
Relative cell number
1.5
1.0
0.5
0.0
**
***
shCtrl
shACLY
shOGDH
c
shControl
shACLY
shOGDH
shACLY + shOGDH
CAOV3
HeyA8
d
Ctrl
OGDH
ACLY
β-Actin
e
HeyA8
CAOV3
Relative cell number
1.5
1.0
0.5
0.0
**
***
ns
shCtrl
shUSP13
Ctrl
ACLY
OGDH
f
shControl
shUSP13
Ctrl
ACLY+OGDH
ACLY
OGDH
CAOV3
HeyA8

Supplementary Figure 10. ACLY and OGDH are essential targets for the function of USP13 in tumourigenesis. (**a**) Knockdown of ACLY or OGDH in CAOV3 cells was examined by western blot analysis. (**b**) Relative proliferation of HeyA8 or CAOV3 cells stably expressing control, ACLY or/and OGDH shRNAs. The non-silencing scramble shRNA was used as a negative control. (**c**) Representative images of tumours in each group included in (**b**). (**d**) Restoration of ACLY and OGDH expression in USP13-knockdown CAOV3 cells. (**e**) Exogenous expression of OGDH or/and ACLY rescued the proliferation of HeyA8 and CAOV3 cells with USP13 knockdown. Ctrl: empty expression vector. (**f**) Representative images of tumours included in each group in (**e**). Scale bar: 1 cm. In this figure, error bars represent ± SD. ns: non-significant. *p<0.05, ** p<0.01, *** p<0.001.

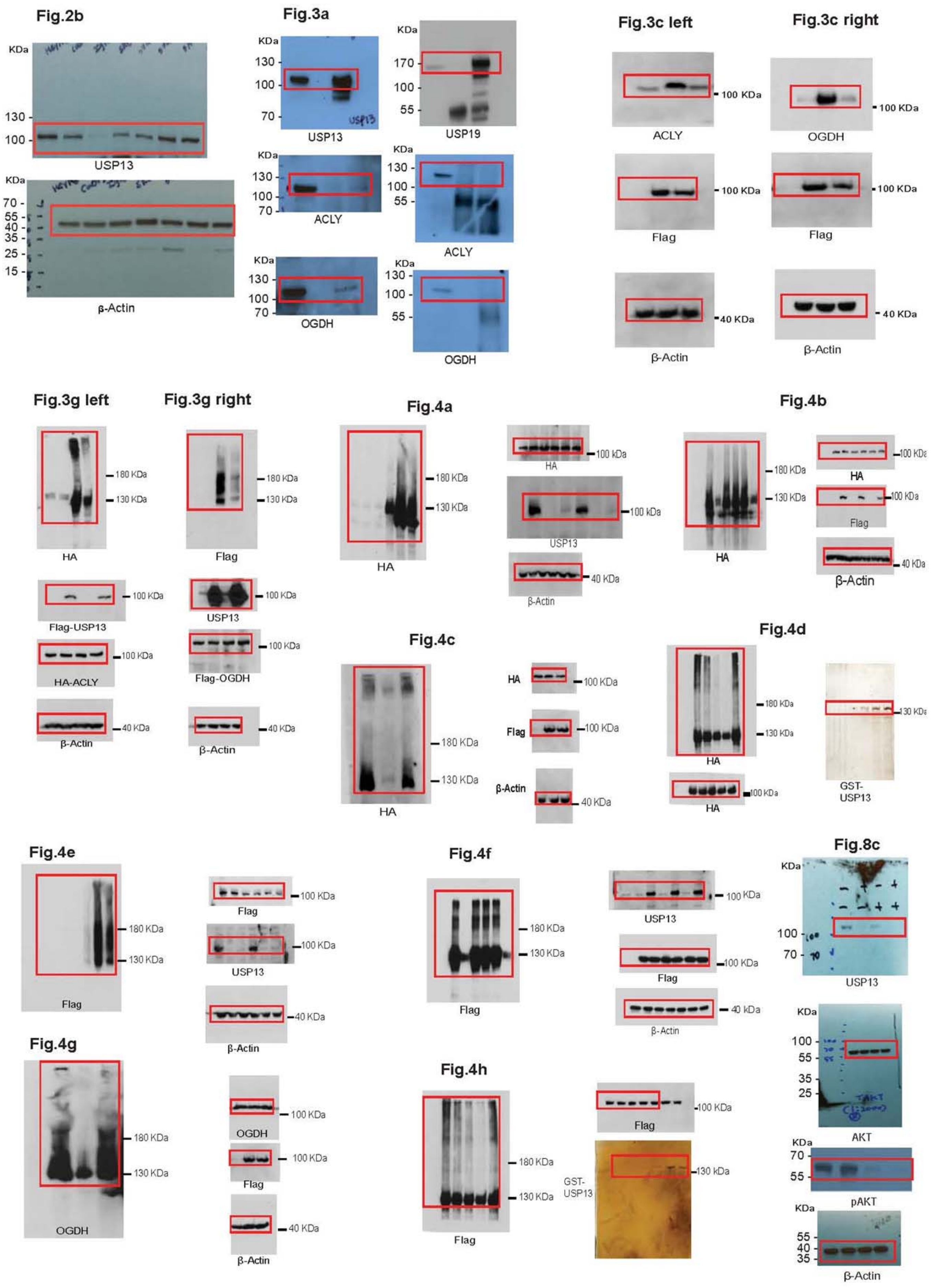

Supplementary Figure 11. Uncropped scans of the most important western blots.
